# Preeclampsia knowledge among postpartum women treated for preeclampsia and eclampsia at Korle Bu Teaching Hospital in Accra, Ghana

**DOI:** 10.1186/s12884-020-03316-w

**Published:** 2020-10-15

**Authors:** Avina Joshi, Titus Beyuo, Samuel A. Oppong, Cheryl A. Moyer, Emma R. Lawrence

**Affiliations:** 1 University of Massachusetts Medical School, 55 N. Lake Ave, Worcester, MA 01655 USA; 2 University of Ghana School of Medicine and Dentistry, Slater Avenue, Accra, Ghana; 3 Department of Obstetrics & Gynaecology, Korle Bu Teaching Hospital, Guggisberg Avenue, Accra, Ghana; 4 Global REACH, University of Michigan Medical School, 1301 Catherine St, Ann Arbor, MI 48109 USA; 5 Department of Obstetrics & Gynecology, University of Michigan Medical School, 1500 E. Medical Center Dr, Ann Arbor, MI 48109 USA

**Keywords:** Maternal health, Pregnancy, Preeclampsia, Eclampsia, Patient knowledge, Patient education, Provider counseling, Sub-Saharan Africa

## Abstract

**Background:**

Preeclampsia/eclampsia is a major cause of maternal morbidity and mortality worldwide, yet patients’ perspectives about their diagnosis are not well understood. Our study examines patient knowledge among women with preeclampsia/eclampsia in a large urban hospital in Ghana.

**Methods:**

Postpartum women diagnosed with preeclampsia or eclampsia were asked to complete a survey 2–5 days after delivery that assessed demographic information, key obstetric factors, and questions regarding provider counseling. Provider counseling on diagnosis, causes, complications, and future health effects of preeclampsia/eclampsia was quantified on a 4-point scale (‘Counseling Composite Score’). Participants also completed an objective knowledge assessment regarding preeclampsia/eclampsia, scored from 0 to 22 points (‘Preeclampsia/Eclampsia Knowledge Score’ (PEKS)). Linear regression was used to identify predictors of knowledge score.

**Results:**

A total of 150 participants were recruited, 88.7% (133) with preeclampsia and 11.3% (17) with eclampsia. Participants had a median age of 32 years, median parity of 2, and mean number of 5.4 antenatal visits. Approximately half of participants reported primary education as their highest level of education. While 74% of women reported having a complication during pregnancy, only 32% of participants with preeclampsia were able to correctly identify their diagnosis, and no participants diagnosed with eclampsia could correctly identify their diagnosis. Thirty-one percent of participants reported receiving no counseling from providers, and only 11% received counseling in all four categories. Even when counseled, 40–50% of participants reported incomplete understanding. Out of 22 possible points on a cumulative knowledge assessment scale, participants had a mean score of 12.9 ± 0.38. Adjusting for age, parity, and the number of antenatal visits, higher scores on the knowledge assessment are associated with more provider counseling (β 1.4, SE 0.3, *p* < 0.001) and higher level of education (β 1.3, SE 0.48, *p* = 0.008).

**Conclusions:**

Counseling by healthcare providers is associated with higher performance on a knowledge assessment about preeclampsia/eclampsia. Patient knowledge about preeclampsia/eclampsia is important for efforts to encourage informed healthcare decisions, promote early antenatal care, and improve self-recognition of warning signs—ultimately improving morbidity and reducing mortality.

## Background

Preeclampsia and eclampsia are leading causes of maternal morbidity and mortality [1]. The burden of preeclampsia and eclampsia is most significant in low- and middle-income countries (LMICs), where hypertensive disorders of pregnancy account for 10–15% of maternal deaths [1, 2]. In many LMICs, including the West African country of Ghana, hypertensive disorders of pregnancy have overtaken hemorrhage as the leading cause of maternal mortality [3, 4].

In pregnancies complicated by preeclampsia and eclampsia, improved outcomes are seen with early identification of symptoms, prompt presentation to healthcare facilities, and subsequent management with antihypertensive medications, magnesium sulfate, and delivery of the fetus and placenta [2, 5–8]. Development of preeclampsia or eclampsia is a significant risk factor for recurrence in subsequent pregnancies [7]. Since preeclampsia and eclampsia are exclusively complications of pregnancy, antenatal care (ANC) visits and intrapartum admission are important opportunities for patient counseling [9, 10]. Prenatal education on symptoms of preeclampsia and eclampsia may result in improved outcomes [11–14], with studies linking understanding of counseling to higher rates of women taking action and reporting symptoms [15].

Despite the important connections between women’s knowledge of warning signs and seeking appropriate care, little research has addressed the patient perspective. Studies conducted in high-income countries demonstrate that only half of patients were counseled on signs and symptoms of preeclampsia [16], even though counseling by healthcare providers is associated with increased patient knowledge [16–18].

In LMICs, healthcare providers and patients face unique challenges, including lower general education levels and health literacy, and limited access and utilization of antenatal care services [19, 20]. Previous research in Ghana [17], Tanzania [21, 22], and Malaysia [23] all demonstrate low levels of knowledge about preeclampsia among pregnant women. However, none of these studies focused on women with a clinical diagnosis of preeclampsia or eclampsia.

A better understanding of a patient’s knowledge about her diagnosis and implications for future pregnancies is important when caring for high-risk women. However, the experience and knowledge of women with preeclampsia and eclampsia in LMICs is largely unknown. The current study fills this gap—evaluating counseling, understanding, and knowledge of postpartum women diagnosed with preeclampsia and eclampsia in a large urban tertiary hospital in Ghana.

## Methods

This study took place at the Korle Bu Teaching Hospital (KBTH), Ghana’s largest tertiary care hospital located in the capital city of Accra. The maternity unit serves patients receiving antenatal care at KBTH and referral cases from the southern half of the country, with approximately 9500 deliveries per year.

Ethical approval was granted by the Scientific and Technical Committee of the Korle Bu Teaching Hospital (KBTH-IRB 00096/2018) and the University of Michigan Institutional Review Board (HUM00139104). Study participants were identified through the ongoing MOPEP Trial, a randomized controlled trial of comparative dosing regimens of magnesium sulfate for management of preeclampsia and eclampsia [24]. Inclusion criteria were admission to KBTH with a diagnosis of eclampsia or preeclampsia with severe features, age 18 years or older, and fluency in English or Twi/Akan.

Data collection was completed between November 2019 and March 2020 by two research assistants, one of whom was fluent in Twi/Akan. Eligible participants were recruited in the postpartum inpatient ward at least 2 days after delivery, and a written informed consent process was completed. A standardized survey was verbally administered by a research assistant. Surveys were completed at the bedside in the language choice of the participant. See Additional file 1 for the complete survey questions.

Demographic information and obstetric history were collected from the participants’ clinical charts. The survey consisted of two parts. Part I (24 questions) focused on patient perceptions of provider counseling about their clinical diagnosis. This section assessed the recollection and comprehension of information provided by the healthcare provider on four counseling categories: diagnosis, causes, possible complications, and future health effects, including likelihood of recurrence in future pregnancies. A counseling composite score was created ranging from 0 to 4 possible points, where one point was awarded for a participant responding ‘Yes’ to being counseled on any of the four categories. Participants who responded ‘Yes’ to being counseled on any of these four categories were then asked a follow-up question regarding their perceived level of understanding of the counseling. Understanding was graded on a 4-point scale: None, Some, Most, or All. The interviewer explained that ‘Some’ meant understanding less than half of the information provided, while ‘Most’ meant understanding more than half. During data analysis, ‘Less than 50% Understanding’ was defined as a response of ‘None’ or ‘Some’ and ‘More than 50% Understanding’ was defined as a response of ‘Most’ or ‘All.’

Part II (10 questions) was an objective knowledge assessment, adapted to the local Ghanaian context from a survey developed by the Preeclampsia Foundation [16]. Participants were asked multiple choice and true/false questions about risk factors, symptoms, and management of preeclampsia. Responses were summed to generate a cumulative Preeclampsia/Eclampsia Knowledge Score (PEKS), with a total of 22 possible points. This cumulative knowledge score was used as the primary outcome variable.

Surveys were completed via pen and paper, entered into REDCap, and downloaded into STATA (Version 16.0 StataCorp. 2019) for cleaning and analysis. Descriptive statistics were calculated for all key variables using medians (minimum/maximum range) and frequencies (proportion). Bivariate linear regression analysis was used to evaluate the relationship between the PEKS, demographic and clinical factors, and counseling indicators. Significant variables in our bivariate model were included in a multivariate linear regression analysis, which was also adjusted by age, parity, and number of antenatal visits, as these are often linked to knowledge of pregnancy-related factors. All tests were two-tailed and a *p* value of < 0.05 was accepted as significant.

## Results

**Fig. 1:**
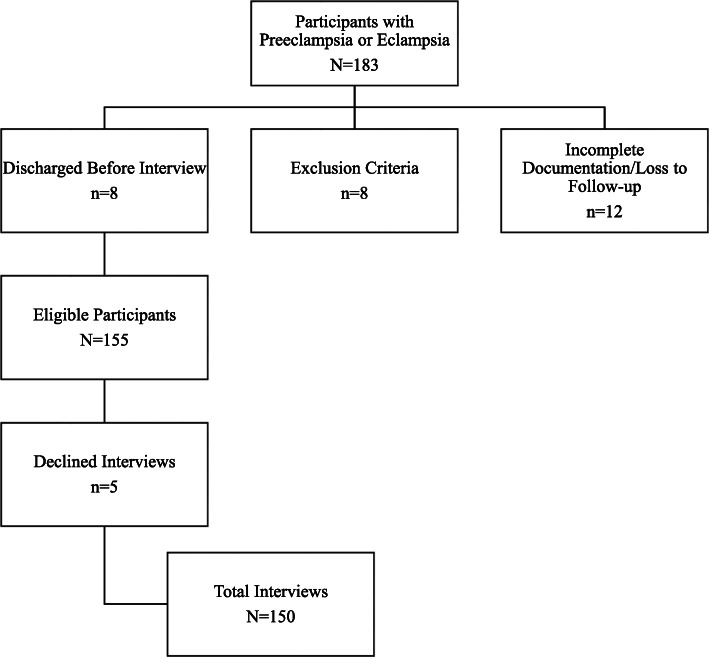
Participant Recruitment Flow Chart

From November 2019–March 2020, a total of 150 participants completed the study (Fig. 1). Table 1 illustrates participant demographics. Participants had a median age of 32 years (range 18–47) and 63.3% (95) were multiparous. Approximately half (70) of participants reported their highest completed level of education as ‘Primary.’ A majority (133, 88.7%) of participants were diagnosed with preeclampsia, while 17 (11.3%) participants were diagnosed with eclampsia. Regarding history of hypertensive disorders of pregnancy, 33 (22.0%) participants had a comorbid diagnosis of chronic hypertension, 10 (6.7%) had preeclampsia/eclampsia in a prior pregnancy, and 11 (7.3%) had gestational hypertension in a prior pregnancy. Most women received care from a midwife (90, 60.4%), attended four or more antenatal visits (108, 72%), and conducted their healthcare communication primarily in Twi/Akan (117, 78%).

**Table 1:** Demographic Factors

Characteristic	Participants (*n* = 150)
Age, years^a^	32 (18–47)
Participant Reported Language Used for Healthcare	
English	33 (22.0)
Twi	117 (78.0)
Highest Level of Completed Education	
None	1 (0.7)
Primary	70 (47.0)
Secondary	39 (26.2)
Tertiary	39 (26.2)
Clinical Diagnosis	
Preeclampsia	133 (88.7)
Eclampsia	17 (11.3)
Parity^a^	2 (1–10)
Primiparous	55 (36.7)
Multiparous	95 (63.3)
Primary Caregiver During Pregnancy	
Specialist obstetrician/gynecologist	42 (29.2)
Medical officer (non-obstetrician)	9 (6.0)
Midwife	90 (60.4)
Other	1 (0.7)
None	7 (4.7)
Number of Antenatal Appointments Attended^a^	5.3 (0–14)
0–3	42 (28.0)
≥ 4	108 (72.0)
Diagnosis of Chronic Hypertension (index pregnancy)	
Yes	33 (22.0)
No	117 (78.0)
Previous Hypertensive Disorder of Pregnancy (previous pregnancies)	
Preeclampsia/Eclampsia	10 (6.7)
Gestational hypertension	11 (7.3)

Data presented as n (%) unless otherwise noted

^a^Median (range: minimum value – maximum value)

**Fig. 2:**
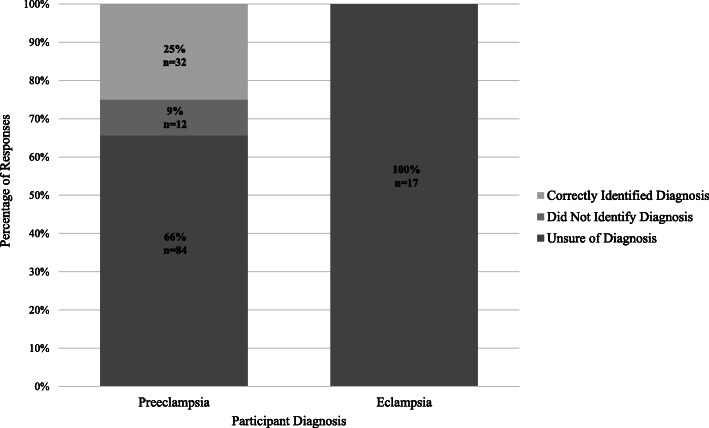
Participants’ Ability to Correctly Identify Diagnosis

Figure 2 illustrates participant responses regarding their understanding of their diagnosis. Only 24% of participants with preeclampsia correctly identified their diagnosis, and none of the participants with eclampsia were able to correctly identify their diagnosis. Additionally, 86 (61.4%) participants reported never hearing about preeclampsia/eclampsia during their pregnancy. While 74% of participants correctly identified the severity of preeclampsia/eclampsia as very serious, almost two-thirds of participants (92, 62%) said they do not understand it well enough to explain it to another person and 73% (108) said they do not know what to do in future pregnancies to prevent the condition or improve its outcome (Table 2).

**Table 2:** Knowledge Assessment

Assessment	Participants (*n* = 150)
Do you feel like you understand your condition well enough to explain it to someone else?	
Yes	57 (38.3)
No	75 (50.3)
I don’t know	17 (11.4)
Do you know what to do in future pregnancies to prevent this condition or improve upon its outcome?	
Yes	40 (27.0)
No	84 (56.8)
I don’t know	24 (16.2)
Who helped you the most with understanding your condition?	
Doctor	54 (36.7)
Midwife	32 (21.8)
Nurse	3 (2.0)
Family member	8 (5.4)
Other	5 (3.4)
None of the above	45 (30.6)
When did you first hear about preeclampsia?	
During pregnancy	54 (38.6)
Month of pregnancy^a^	6.65 ± − 1.74 (2–9)
I never heard about it	86 (61.4)
How serious of a health issue do you think preeclampsia is?	
Not at all serious	5 (3.5)
Somewhat serious	26 (17.9)
Very serious	49 (33.8)
Extremely serious, even life-threatening	62 (42.8)
I don’t know	3 (2.1)
Preeclampsia/Eclampsia Knowledge Score^a^	13.1 ± −4.5 (2–21)

Data presented as n (%) unless otherwise noted

Mean ± SD (Range: minimum value – maximum value)

**Fig. 3:**
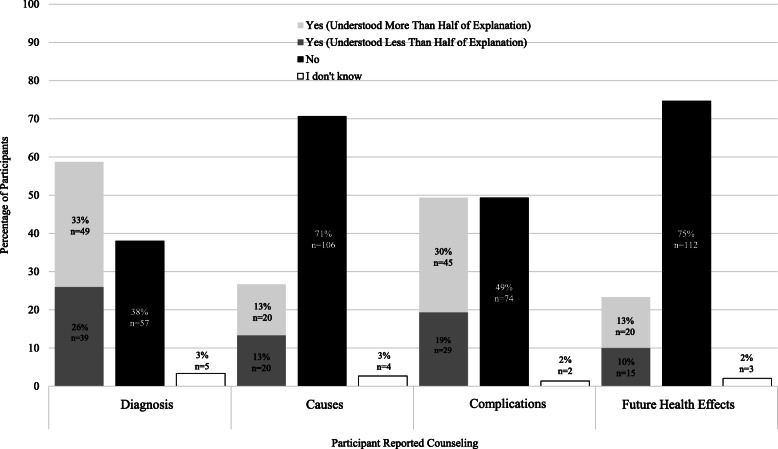
Reported Counseling on Diagnosis, Causes, Complications, and Future Health Effects

**Fig. 4:**
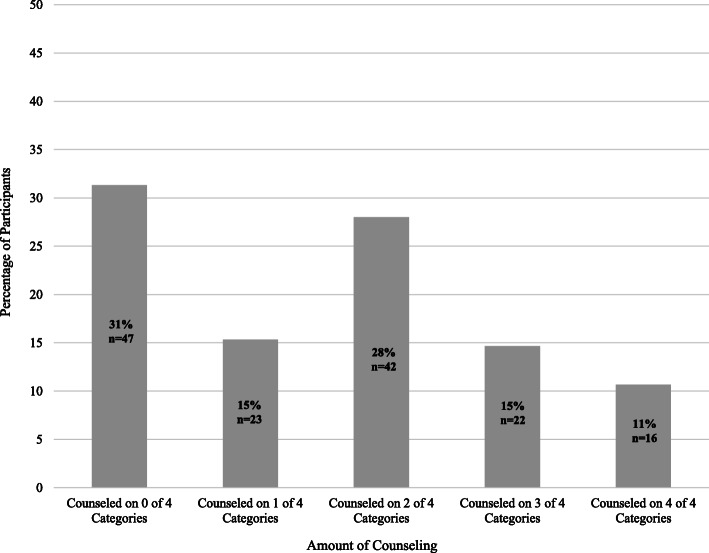
Reported Amount of Provider Counseling on Diagnosis, Causes, Complications and Future Health Effects (Counseling Composite Score)

Figure 3 shows participant perceptions of provider counseling. Eighty-eight (58.7%) women said they received an explanation from a healthcare provider about their diagnosis, 40 (26.7%) about causes of the condition, 74 (49.3%) about complications, and 35 (23.3%) about future health effects. Of those who reported receiving counseling on these topics, 44.0% of women who received information about their diagnosis, 50% who received information about the causes, 39.2% who received information about potential complications, and 42.9% who received information about future health effects said they understood less than half of information provided. Figure 4 illustrates the ‘Counseling Composite’ Score for participants. The largest proportion of participants (47, 31.3%) did not receive counseling on any of the four categories.

**Fig. 5:**
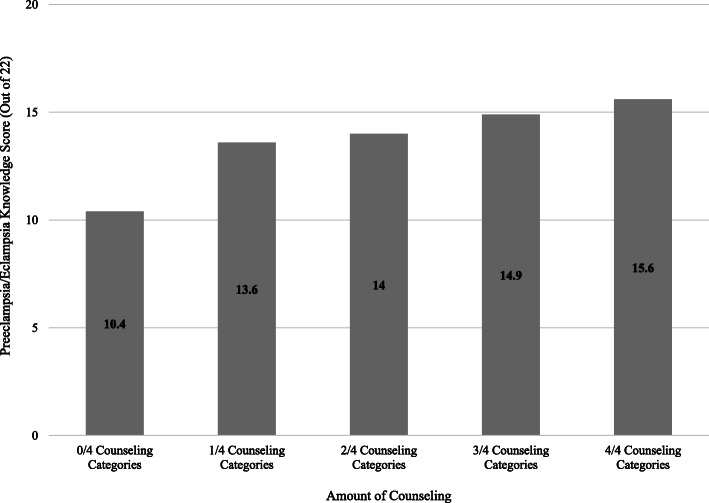
Impact of Provider Counseling on Preeclampsia/Eclampsia Knowledge Assessment Score

Out of 22 possible points on the PEKS, participants scored a mean of 13 (SD 4.5) with a range of 2–21. Table 3 demonstrates bivariate linear regression, evaluating the relationship between the PEKS and demographics, obstetric factors, and perceived level of counseling. Variables significantly associated with PEKS included language used for healthcare, level of education, comorbid chronic hypertension, and history of preeclampsia/eclampsia in a previous pregnancy. Patient-reported provider counseling in each of the four categories, as well as the composite counseling score, were also significant. After adjusting for age, parity, and number of attended antenatal visits, higher level of education and a higher counseling composite score were significant contributors to a participant’s PEKS (Table 4). Each level of increasing education— no education, primary, secondary, and tertiary—was associated with an increase of 1.3 points on the PEKS (β 1.3, SE 0.48, *p* = 0.008). Each increase of one point on the counseling composite score was associated with an increase of 1.4 points on the PEKS (β 1.4, SE 0.3, *p* < 0.001). Compared to participants who did not receive any counseling, participants counseled on all four domains scored on average 5.2 points higher on the knowledge score (50% increase). Figure 5 demonstrates the knowledge score for participants at each level of the counseling composite score, adjusted by the other variables in our final model.

**Table 3:** Preeclampsia/Eclampsia Knowledge Score Bivariate Analysis

Factor	Preeclampsia/Eclampsia Knowledge Score			
	Mean ± SD	β	95% CI	*p*-value
Demographic Characteristics				
Age, years				
< 20	5.60 ± −3.65	−8.95	−12.95 – − 4.95	< 0.001
20–24	12.01 ± − 3.37	−2.49	− 4.88 – − 0.11	0.041
25–29	12.83 ± − 3.98	−1.69	− 3.65 – − 0.26	0.088
30–34	14.54 ± − 4.35	REF	REF	REF
35–39	12.63 ± − 4.77	−1.91	− 3.77 – − 0.06	0.044
≥ 40	14.9 ± − 4.51	0.35	−2.62 – 3.33	0.815
Main Language Used for Healthcare				
English	15.21 ± −3.11	2.75	1.03 – 4.47	0.002
Twi	12.46 ± 4.70	REF	REF	REF
Highest Level of Completed Education				
None	7.00 ± 0.00	−4.61	−13.12 – 3.88	0.285
Primary	11.61 ± −4.75	REF	REF	REF
Secondary	13.49 ± −4.23	1.87	0.19–3.56	0.030
Tertiary	15.49 ± − 3.27	3.87	2.18–5.56	< 0.001
Clinical Diagnosis				
Preeclampsia	13.18 ± −4.39	REF	REF	REF
Eclampsia	12.18 ± −5.64	−1.00	− 3.32 – 1.31	0.392
Parity^a^				
Primiparous	12.31 ± − 4.39	REF	REF	REF
Multiparous	13.5 ± −4.36	1.20	−0.32 – 2.71	0.120
Primary Caregiver During Pregnancy				
Specialist obstetrician/gynecologist	14.14 ± −4.15	1.54	−0.04 – 3.12	0.056
Medical officer (non-obstetrician)	17.33 ± −2.24	4.73	1.78–7.69	0.002
Midwife	12.60 ± −4.37	REF	REF	REF
Other	10.0 ± 0.00	−2.60	−11.11 – 5.91	0.547
None	8.00 ± 5.63	−4.60	−7.92 – − 1.28	0.007
Number of Antenatal Appointments Attended^a^				
0–3	12.69 ± −5.29	REF	REF	REF
≥ 4	13.21 ± − 4.23	0.52	−1.11 – 2.16	0.529
Diagnosis of Chronic Hypertension				
Yes	14.88 ± −3.71	2.32	0.59–4.06	0.009
No	12.56 ± −4.63	REF	REF	REF
Previous Preeclampsia/Eclampsia				
Yes	16.10 ± −4.04	3.25	0.35–6.15	0.028
No	12.85 ± −4.51	REF	REF	REF
Previous Gestational Hypertension				
Yes	12.91 ± −4.50	−0.17	−2.99 – 2.65	0.905
No	13.08 ± −4.56	REF	REF	REF
Previous Hypertensive Disorder of Pregnancy				
Yes	14.43 ± −4.49	1.58	−0.52 – 3.69	0.139
No	12.84 ± −4.53	REF	REF	REF
Provider Counseling				
Did your health caregiver provide information about your diagnosis?				
Yes	14.65 ± −3.56	3.83	2.47–5.18	< 0.001
No	10.82 ± −4.85	REF	REF	REF
Did your health caregiver provide information about your causes?				
Yes	15.38 ± −3.34	3.15	1.57–4.73	< 0.001
No	12.23 ± −4.64	REF	REF	REF
Did your health caregiver provide information about your complications?				
Yes	14.92 ± −2.99	3.66	2.31–5.00	< 0.001
No	11.26 ± −5.05	REF	REF	REF
Did your health caregiver provide information about your future health effects?				
Yes	15.54 ± −2.98	3.23	1.57–4.89	< 0.001
No	12.31 ± −4.67	REF	REF	REF
Provider Counseling Composite Score				
Counseled on 0 of 4 components	9.78 ± −4.93	REF	REF	REF
Counseled on 1 of 4 components	13.52 ± −4.04	3.73	1.76–5.71	REF
Counseled on 2 of 4 components	14.00 ± −3.45	4.21	2.57–5.86	REF
Counseled on 3 of 4 components	15.41 ± −2.44	5.62	3.62–7.62	REF
Counseled on 4 of 4 components	16.38 ± −3.01	6.59	4.35–8.83	REF

**Table 4:** Preeclampsia/Eclampsia Knowledge Score Multivariate Analysis

Model^a^	β	95% CI	*p*-value
Age	0.05	−0.08 – 0.17	0.453
Language of Healthcare	−0.25	−2.12 – 1.62	0.790
Education Level	1.29	0.34–2.23	0.008
Parity	0.24	−0.22 – 0.71	0.306
Antenatal Care (category)	0.36	−1.03 – 1.75	0.609
Diagnosis of Chronic Hypertension	1.29	−0.25 – 2.83	0.101
Previous Preeclampsia/Eclampsia	0.77	−1.77 – 3.31	0.550
Counseling Composite Score	1.35	0.87–1.84	< 0.001

^a^R squared = 0.36

## Discussion

Our study explores the patient perspective of knowledge and counseling on preeclampsia/eclampsia in an urban LMIC setting. Although 74% of women recognized having a complication during their pregnancy, one-third of women reported receiving no counseling from a provider regarding their condition. The biggest gap in counseling appears to be counseling on causes of the condition, with more than two-thirds of participants reporting no counseling on causes. Even when women reported being counseled, a large proportion reported understanding less than half of the information provided. Seventy-three percent of participants reported not knowing what to do to prevent or improve their condition in future pregnancies. Out of 22 possible points on the knowledge assessment, the average knowledge score was 13. Our multivariate analysis demonstrated that after controlling for age, parity, and number of antenatal visits, a higher knowledge score was predicted by a higher level of education and an increased amount of direct provider counseling.

Consistent with findings from studies of pregnant women in the United States [16], elsewhere in Ghana [17], and in other LMICs [21–23], our study demonstrates a low level of knowledge about preeclampsia/eclampsia. In the United States, 57% of participants reported being counseled on signs and symptoms of preeclampsia/eclampsia [16], compared to only 49% in our Ghanaian population. Importantly, our study population consisted of women with a recent clinical diagnosis of preeclampsia or eclampsia undergoing inpatient management of this complication of pregnancy. It is especially imperative for this particular population to have an adequate level of knowledge and understanding, as the condition has directly impacted their just completed pregnancies, may continue to impact their health in the postpartum period, and is more likely to recur in their future pregnancies. Our study demonstrated a significant relationship between provider counseling on preeclampsia and participants’ knowledge score. This key relationship has not been extensively explored, but agrees with findings from the United States [16, 18]. Education level was also a significant predictor of knowledge score, which is concordant with other studies performed in Ghana [17] and the United States [18]. Other studies demonstrated that higher literacy, multiparity, and a history of preeclampsia in a prior pregnancy were predictive of knowledge scores [18]. These relationships were significant in our bivariate analysis, but were no longer significant in our adjusted final model.

In 2016, updated World Health Organization (WHO) guidelines increased the number of recommended antenatal visits from four to eight, with the goal of better preventing and managing pregnancy-related or concurrent disease and providing health education [25]. Of note, our study demonstrated that the number of attended antenatal visits did not correlate with a higher PEKS score. While direct provider counseling increased a participant’s PEKS score, more frequent antenatal visits did not. This finding suggests that while increasing the frequency of antenatal visits may be important for many reasons, addressing systemic barriers to effective patient-provider communication, education, and counseling is important to see meaningful change in patient knowledge. Regarding ANC attendance and patient knowledge, our study fills a gap in the literature, as there are few studies that examine women’s knowledge of preeclampsia and its correlation to the number of antenatal care visits, especially when examined as a continuous variable in linear regression. Within sub-Saharan Africa, studies show that patient education level is linked to increased knowledge regarding preeclampsia [17] and birth preparedness and complication readiness [26]. One study concluded that ANC attendance increased participant knowledge of obstetric danger signs during pregnancy and childbirth by approximately 2.5 times; however, this study treated ANC attendance as a binary yes/no variable, preventing the examination of a dose-response relationship between the number of ANC visits and knowledge. Additionally, this study demonstrated that most participants were only able to identify vaginal bleeding as an obstetric warning sign, while less than half were able to identify any of the symptoms of preeclampsia as an obstetric warning sign [27]. This finding is consistent with another study that demonstrated less than one-third of participants could identify preeclampsia-specific warning signs [26]. This suggests that current ANC practices may not provide education and counseling that is comprehensive of all dangerous pregnancy-related complications. Addressing this problem requires a multidisciplinary approach and patients may benefit from other WHO-recommended methods of antenatal education such as group antenatal visits and community-based education [25].

Our study fills an important gap in the literature by exploring multiple predictors of patient knowledge, evaluating patient comprehension of provider counseling, and assessing the role of counseling in patient knowledge of preeclampsia in a LMIC setting. Strengths of the study include being embedded within a larger randomized controlled trial, which allowed our study population to consist entirely of women whose recent pregnancies were complicated by preeclampsia or eclampsia. To our knowledge, this is the first study of its kind to assess knowledge in this key targeted population. Participant knowledge of preeclampsia was assessed using a previously validated objective assessment created by the Preeclampsia Foundation [16], modified to the local context after extensive pilot testing. Although performed at a single site, the Korle Bu Teaching Hospital provides care for a wide range of attendants and referral patients from Ghana’s capital city of Accra, as well as surrounding peri-urban and rural areas—supporting generalizability across Ghana. Diversity of participants is reflected in the range of age, language, education level, and number of ANC visits represented by our sample.

Limitations include challenges with language and translation, particularly because there is no direct Twi/Akan translation of “preeclampsia” or “eclampsia.” A pilot period, with feedback from patients and healthcare providers, was utilized to standardize translation of English questions into Twi/Akan. However, nuanced differences in translation may persist, causing bias between participants who completed the survey in English versus in Twi/Akan. Survey questions were verbally presented by a research assistant in the participant’s language of choice to minimize limitations with literacy. Interviews were completed in an inpatient hospital setting, with potential for participants to be hesitant to respond negatively about counseling from their healthcare providers. However, research assistants had no role in patient care and the informed consent process outlined standards of confidentiality and anonymity. Additional limitations include recall bias, where participants with higher health literacy and more knowledge about preeclampsia may recall that more provider counseling was performed. Recall bias was minimized by not disclosing correct responses to the knowledge questions until the entire survey was complete. Additionally, recall bias could have unequally affected patients diagnosed with eclampsia, especially regarding provider counseling during antenatal and pre-delivery care. Lastly, additional studies are required to assess retention of knowledge over time and changes in knowledge after a patient’s outpatient postpartum visit.

## Conclusions

Our study highlights the importance of provider-based counseling in improving knowledge about preeclampsia. We demonstrate that average knowledge about preeclampsia is low, and increased counseling by healthcare providers is associated with higher knowledge scores. Knowledge about preeclampsia is important so patients may identify warning symptoms of new or worsening disease, improve healthcare-seeking behavior, and make informed healthcare decisions [15]. Given significant risk of recurrence in subsequent pregnancies, patient knowledge about causes, prevention, and recurrence of preeclampsia can promote early prenatal visits and hospital deliveries for these high-risk women. While we acknowledge there are many systemic barriers that can make counseling difficult for providers, improving counseling and ensuring that patients understand their diagnosis of preeclampsia/eclampsia is likely to improve outcomes. Findings from this research have significant implications for developing educational interventions to address knowledge gaps and improve patient counseling. Additional research is needed to evaluate the impact of educational interventions on patient knowledge, and to explore the relationship between patient knowledge and maternal and neonatal outcomes.

## Supplementary information

**Additional file 1: Survey questions**. Part I (24 questions) and Part II (10 questions) of the survey verbally administered to study participants.

## Data Availability

The datasets used and/or analyzed during the current study are available from the corresponding author on reasonable request.

## Abbreviations

PEKS: Preeclampsia/Eclampsia Knowledge Score
LMIC: Low- and middle-income country
ANC: Antenatal care
KBTH: Korle Bu Teaching Hospital
WHO: World Health Organization.
