# Supplementary material for: Preeclampsia knowledge among postpartum women treated for preeclampsia and eclampsia at Korle Bu Teaching Hospital in Accra, Ghana
Source: BMC Pregnancy Childbirth. 2020 Oct 15;20:625. doi: 10.1186/s12884-020-03316-w (PMC7566025; doi:10.1186/s12884-020-03316-w)
Supplement: Supplementary file 1 — Additional file 1: Survey questions. Part I (24 questions) and Part II (10 questions) of the survey verbally administered to study participants. [file 12884_2020_3316_MOESM1_ESM.docx]

**Additional file 1. Survey questions**

Part I Survey (Counseling Composite Score):

1. Did your health caregiver provide you information about your diagnosis? (Answer Choices: Yes, No, I don’t know)
2. If yes, how much of this information about the diagnosis did you understand? (Answer choices: None, Some, Most, All)
3. Did your health caregiver provide information about the causes of your condition? (Answer Choices: Yes, No, I don’t know)
4. If yes, how much of this information about the causes did you understand? (Answer choices: None, Some, Most, All)
5. Did your health caregiver provide information about possible complications and effects of your condition on your just ended pregnancy? (Answer Choices: Yes, No, I don’t know)
6. If yes, how much of this information about the possible complications did you understand? (Answer choices: None, Some, Most, All)
7. Did your health caregiver provide information about future health effects of your condition? (Answer choices: Yes, No, I don’t know)
8. If yes, how much of this information about future health effects did you understand? (Answer choices: None, Some, Most, All).

Part II Survey (Components of the Preeclampsia/Eclampsia Knowledge Score):

1. How serious of a health issue do you think preeclampsia is? (Answer choices: Not at all serious, Somewhat serious, Very serious, Extremely serious – even life threatening)
2. Below is a list of symptoms that may or may not be warning signs for preeclampsia. For each of the following, indicate if you think the listed symptom, when it occurs with high blood pressure, is a warning sign for preeclampsia. (Per each symptom, answer choices: Yes, No, I don’t know)
   1. Headache
   2. Seeing spots/flashing lights/losing all or part of your eyesight
   3. Uterine contractions
   4. Back pain
   5. Chest pain
   6. Sleepiness
   7. Pain with urination
   8. Upper abdominal pain
3. Below is a list of statements about preeclampsia. Please indicate if you think each is true, false or you are unsure. (Per each statement, answer choices: True, False, I don’t know).
   1. High blood pressure is a sign of preeclampsia.
   2. Any pregnant woman, even a healthy one, is at risk for preeclampsia.
   3. Women with preeclampsia are at risk of having a premature baby.
   4. Women with preeclampsia are at greater risk of having a stroke.
   5. Women with preeclampsia are at greater risk of having a baby die before it is born.
   6. Women with preeclampsia are at greater risk of having a seizure.
   7. Delivery is the cure for preeclampsia.
   8. Women with preeclampsia are at a greater risk for future health problems.
   9. Preeclampsia can occur up to 6 weeks after delivery.
   10. Women with preeclampsia need to measure their blood sugars every day.
   11. Women with preeclampsia are at risk of having a large baby.
   12. Only women who are overweight are at risk for preeclampsia.
   13. Women who have had preeclampsia in a previous pregnancy are at greater risk for having preeclampsia again.
4. Which of the following actions do you think would be appropriate to take if you have symptoms associated with preeclampsia? (Participants could select as many options as they deemed correct)
   1. Call my doctor or midwife
   2. Go to the hospital
   3. Lie down
   4. Drink a glass of water
   5. Take medicine for pain at home
   6. Wait one day to see if the symptoms get better
   7. Go to church/mosque/shrine/a prayer camp
   8. See an herbalist/traditional healer
   9. Other
5. Indicate which of the following option(s) you believe are treatments for preeclampsia. (Participants could select as many options as they deemed correct)
   1. Regular blood sugar readings
   2. Blood pressure medications
   3. Pain medications
   4. Blood Transfusion
   5. Magnesium Sulfate Injection
   6. Treatment for Infection
   7. Delivery of the baby
   8. Herbal preparations
   9. Other
